# 2-(4-Fluoro­phen­yl)-3-isopropyl­sulfinyl-5,6-methyl­enedi­oxy-1-benzofuran

**DOI:** 10.1107/S1600536811051178

**Published:** 2011-11-30

**Authors:** Pil Ja Seo, Hong Dae Choi, Byeng Wha Son, Uk Lee

**Affiliations:** a Department of Chemistry, Dongeui University, San 24 Kaya-dong Busanjin-gu, Busan 614-714, Republic of Korea; b Department of Chemistry, Pukyong National University, 599-1 Daeyeon 3-dong, Nam-gu, Busan 608-737, Republic of Korea

## Abstract

In the title compound, C_18_H_15_FO_4_S, the fluoro­benzene ring makes a dihedral angle of 4.3 (1)° with the mean plane of the benzofuran fragment. In the crystal, mol­ecules are linked by weak inter­molecular C—H⋯O hydrogen bonds. The O atom of the sulfinyl group is disordered over two orientations, with site-occupancy factors of 0.940 (3) and 0.060 (3).

## Related literature

For the pharmacological activity of benzofuran compounds, see: Aslam *et al.* (2009 ▶); Galal *et al.* (2009 ▶); Khan *et al.* (2005 ▶). For natural products with benzofuran rings, see: Akgul & Anil (2003 ▶); Soekamto *et al.* (2003 ▶). For the crystal structures of related compounds, see: Choi *et al.* (2010**a* ▶,b* ▶).
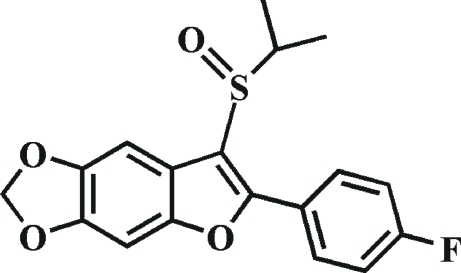


## Experimental

### 

#### Crystal data

C_18_H_15_FO_4_S
*M* *_r_* = 346.36
Triclinic, 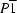

*a* = 6.2519 (1) Å
*b* = 9.6773 (2) Å
*c* = 12.9267 (2) Å
α = 90.122 (1)°
β = 94.726 (1)°
γ = 101.920 (1)°
*V* = 762.47 (2) Å^3^
*Z* = 2
Mo *K*α radiation
μ = 0.24 mm^−1^
*T* = 173 K
0.45 × 0.21 × 0.14 mm

#### Data collection

Bruker SMART APEXII CCD diffractometer
Absorption correction: multi-scan (*SADABS*; Bruker, 2009 ▶) *T*_min_ = 0.898, *T*_max_ = 0.967
13552 measured reflections
3488 independent reflections
3171 reflections with *I* > 2σ(*I*)
*R*_int_ = 0.024

#### Refinement

*R*[*F* ^2^ > 2σ(*F* ^2^)] = 0.039
*wR*(*F* ^2^) = 0.101
*S* = 1.06
3488 reflections
229 parameters
4 restraints
H-atom parameters constrained
Δρ_max_ = 0.56 e Å^−3^
Δρ_min_ = −0.40 e Å^−3^

### 

Data collection: *APEX2* (Bruker, 2009 ▶); cell refinement: *SAINT* (Bruker, 2009 ▶); data reduction: *SAINT*; program(s) used to solve structure: *SHELXS97* (Sheldrick, 2008 ▶); program(s) used to refine structure: *SHELXL97* (Sheldrick, 2008 ▶); molecular graphics: *ORTEP-3* (Farrugia, 1997 ▶) and *DIAMOND* (Brandenburg, 1998 ▶); software used to prepare material for publication: *SHELXL97*.

## Supplementary Material

Crystal structure: contains datablock(s) global, I. DOI: 10.1107/S1600536811051178/fj2487sup1.cif

Structure factors: contains datablock(s) I. DOI: 10.1107/S1600536811051178/fj2487Isup2.hkl

Supplementary material file. DOI: 10.1107/S1600536811051178/fj2487Isup3.cml

Additional supplementary materials:  crystallographic information; 3D view; checkCIF report

## Figures and Tables

**Table 1:** Hydrogen-bond geometry (Å, °)

*D*—H⋯*A*	*D*—H	H⋯*A*	*D*⋯*A*	*D*—H⋯*A*
C5—H5*B*⋯O4*A*^i^	0.99	2.27	3.231 (2)	163
C18—H18*A*⋯O4*A*^ii^	0.98	2.49	3.354 (2)	147

Symmetry codes: (i) 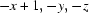; (ii) 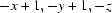.

**Fig. 1.**
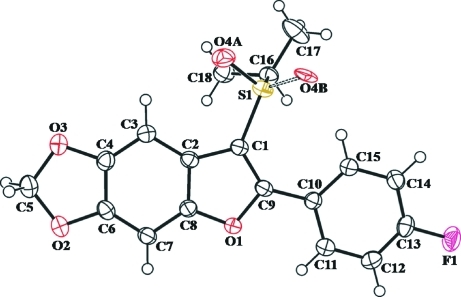
The molecular structure of the title compound with the atom numbering scheme. Displacement ellipsoids are drawn at the 50% probability level. H atoms are presented as small spheres of arbitrary radius. The O atom of the sulfinyl group is disordered over two positions with site-occupancy factors, from refinement of 0.940 (3) (Part A) and 0.060 (3) (part B).

**Fig. 2.**
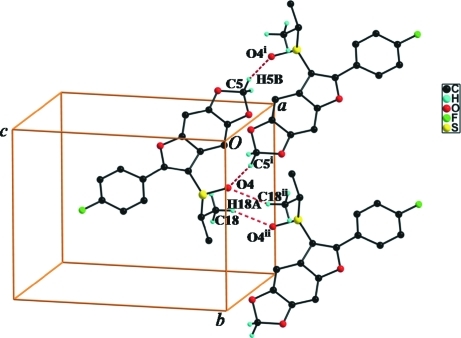
A view of the C—H···O interactions (dotted lines) in the crystal structure of the title compound. H atoms non-participating in hydrogen- bonding were omitted for clarity. [Symmetry codes: (i) - x + 1, - y, - z; (ii) - x + 1, 1- y, - z.]

## supplementary crystallographic information

### Comment

Recently, substituted benzofuran derivatives have drawn much attention due to their valuable pharmacological properties such as antibacterial and antifungal, antitumor and antiviral, and antimicrobial activities (Aslam *et al.*, 2009, Galal *et al.*, 2009, Khan *et al.*, 2005). These benzofuran derivatives occur in a wide range of natural products (Akgul & Anil, 2003; Soekamto *et al.*, 2003). As a part of our ongoing study of 5,6-(methylenedioxy)benzofuran derivatives containing either 3-methylsulfinyl (Choi *et al.*, 2010*a*) or 3-ethylsulfinyl (Choi *et al.*, 2010*b*) substituents, we report herein the crystal structure of the title compound.

In the title molecule (Fig. 1), the benzofuran unit is essentially planar, with a mean deviation of 0.006 (1) Å from the least-squares plane defined by the nine constituent atoms. The dihedral angle formed by the 4-fluorophenyl ring and the mean plane of the benzofuran fragment is 4.3 (1)°. The O atom of the sulfinyl group is disordered over two positions with site-occupancy factors, from refinement, of 0.940 (3)    (Part A) and  0.060 (3)  (part B). The crystal packing is stabilized by weak intermolecular C—H···O hydrogen bonds; the first one between a methylene H atom and the O atom of the S═O unit (Table 1, first entry & Fig. 2), and the second one between a methyl H atom of the isopropyl group and the O atom of the S═O unit (Table 1, second entry & Fig. 2).

### Experimental

77% 3-chloroperoxybenzoic acid (224 mg, 1.0 mmol) was added in small portions to a stirred solution of 2-(4-fluorophenyl)-3-isopropylsulfanyl-5,6-methylenedioxy-1-benzofuran (251 mg, 0.8 mmol) in dichloromethane (30 mL) at 273 K. After being stirred at room temperature for 3h, the mixture was washed with saturated sodium bicarbonate solution and the organic layer was separated, dried over magnesium sulfate, filtered and concentrated at reduced pressure. The residue was purified by column chromatography (hexane–ethyl acetate, 1:2 v/v) to afford the title compound as a colorless solid [yield 71%, m.p. 437–438 K; *R*_f_ = 0.55 (hexane–ethyl acetate, 1:2 v/v)]. Single crystals suitable for X-ray diffraction were prepared by slow evaporation of a solution of the title compound in ethyl acetate at room temperature.

### Refinement

All H atoms were positioned geometrically and refined using a riding model, with  C—H = 0.95 Å for the aryl, 1.00 Å for the methine, 0.99 Å for the methylene, and 0.98 Å for the methyl H atoms. *U*_iso_(H) =1.2*U*_eq_(C) for the aryl, methine, and methylene H atoms, and  1.5*U*_eq_(C) for the methyl H atoms. The O atom of sulfinyl group is disordered over two  positions with site-ccupancy factors, from refinement of 0.940 (3) (part A)  and 0.060  (3) (part B). The distance of S—O  sets was restrained to 0.001 Å using command SADI and DELU.

### Crystal data

**Table:**

C_18_H_15_FO_4_S	*Z* = 2
*M**_r_* = 346.36	*F*(000) = 360
Triclinic, *P*1	*D*_x_ = 1.509 Mg m^−^^3^
Hall symbol: -P 1	Mo *K*α radiation, λ = 0.71073 Å
*a* = 6.2519 (1) Å	Cell parameters from 7633 reflections
*b* = 9.6773 (2) Å	θ = 2.6–27.5°
*c* = 12.9267 (2) Å	µ = 0.24 mm^−^^1^
α = 90.122 (1)°	*T* = 173 K
β = 94.726 (1)°	Block, colourless
γ = 101.920 (1)°	0.45 × 0.21 × 0.14 mm
*V* = 762.47 (2) Å^3^	

### Data collection

**Table:**

Bruker SMART APEXII CCD diffractometer	3488 independent reflections
Radiation source: rotating anode	3171 reflections with *I* > 2σ(*I*)
graphite multilayer	*R*_int_ = 0.024
Detector resolution: 10.0 pixels mm^-1^	θ_max_ = 27.5°, θ_min_ = 1.6°
φ and ω scans	*h* = −7→8
Absorption correction: multi-scan (*SADABS*; Bruker, 2009)	*k* = −12→12
*T*_min_ = 0.898, *T*_max_ = 0.967	*l* = −15→16
13552 measured reflections	

### Refinement

**Table:**

Refinement on *F*^2^	Primary atom site location: structure-invariant direct methods
Least-squares matrix: full	Secondary atom site location: difference Fourier map
*R*[*F*^2^ > 2σ(*F*^2^)] = 0.039	Hydrogen site location: difference Fourier map
*wR*(*F*^2^) = 0.101	H-atom parameters constrained
*S* = 1.06	*w* = 1/[σ^2^(*F*_o_^2^) + (0.0457*P*)^2^ + 0.4198*P*] where *P* = (*F*_o_^2^ + 2*F*_c_^2^)/3
3488 reflections	(Δ/σ)_max_ < 0.001
229 parameters	Δρ_max_ = 0.56 e Å^−^^3^
4 restraints	Δρ_min_ = −0.40 e Å^−^^3^

### Special details

**Table:**

Geometry. All esds (except the esd in the dihedral angle between two l.s. planes) are estimated using the full covariance matrix. The cell esds are taken into account individually in the estimation of esds in distances, angles and torsion angles; correlations between esds in cell parameters are only used when they are defined by crystal symmetry. An approximate (isotropic) treatment of cell esds is used for estimating esds involving l.s. planes.
Refinement. Refinement of F^2^ against ALL reflections. The weighted R-factor wR and goodness of fit S are based on F^2^, conventional R-factors R are based on F, with F set to zero for negative F^2^. The threshold expression of F^2^ > 2sigma(F^2^) is used only for calculating R-factors(gt) etc. and is not relevant to the choice of reflections for refinement. R-factors based on F^2^ are statistically about twice as large as those based on F, and R- factors based on ALL data will be even larger.

### Fractional atomic coordinates and isotropic or equivalent isotropic displacement parameters (Å^2^)

**Table:**

	*x*	*y*	*z*	*U*_iso_*/*U*_eq_	Occ. (<1)
S1	0.15386 (6)	0.36031 (4)	0.14314 (3)	0.02733 (12)	
F1	−0.43070 (18)	0.35212 (13)	0.57633 (8)	0.0438 (3)	
O1	0.31016 (17)	0.12210 (11)	0.37742 (8)	0.0264 (2)	
O2	0.8622 (2)	−0.09128 (13)	0.23234 (10)	0.0377 (3)	
O3	0.8308 (2)	0.03336 (14)	0.08091 (10)	0.0415 (3)	
O4A	0.2282 (2)	0.32964 (14)	0.04204 (9)	0.0358 (4)	0.940 (3)
O4B	−0.0806 (7)	0.3600 (18)	0.1198 (15)	0.030 (4)	0.060 (3)
C1	0.2601 (2)	0.25039 (15)	0.23544 (11)	0.0241 (3)	
C2	0.4209 (2)	0.16817 (15)	0.21565 (12)	0.0245 (3)	
C3	0.5457 (3)	0.15352 (16)	0.13162 (12)	0.0282 (3)	
H3	0.5335	0.2030	0.0687	0.034*	
C4	0.6853 (3)	0.06270 (17)	0.14767 (13)	0.0289 (3)	
C5	0.9256 (3)	−0.07578 (18)	0.12790 (14)	0.0343 (4)	
H5A	1.0874	−0.0506	0.1285	0.041*	
H5B	0.8725	−0.1656	0.0882	0.041*	
C6	0.7051 (3)	−0.01235 (16)	0.23908 (13)	0.0279 (3)	
C7	0.5854 (3)	−0.00188 (16)	0.32195 (12)	0.0278 (3)	
H7	0.5975	−0.0530	0.3841	0.033*	
C8	0.4441 (2)	0.09170 (15)	0.30558 (12)	0.0248 (3)	
C9	0.1973 (2)	0.21924 (15)	0.33327 (12)	0.0247 (3)	
C10	0.0418 (2)	0.26129 (16)	0.39856 (12)	0.0249 (3)	
C11	0.0148 (3)	0.20327 (18)	0.49683 (13)	0.0308 (3)	
H11	0.1053	0.1409	0.5222	0.037*	
C12	−0.1410 (3)	0.23498 (19)	0.55767 (13)	0.0343 (4)	
H12	−0.1584	0.1954	0.6244	0.041*	
C13	−0.2699 (3)	0.32515 (18)	0.51912 (13)	0.0309 (3)	
C14	−0.2463 (3)	0.38780 (18)	0.42458 (13)	0.0311 (3)	
H14	−0.3354	0.4518	0.4010	0.037*	
C15	−0.0895 (3)	0.35560 (17)	0.36425 (12)	0.0294 (3)	
H15	−0.0709	0.3982	0.2986	0.035*	
C16	0.3187 (3)	0.53086 (16)	0.19013 (13)	0.0289 (3)	
H16	0.3011	0.5410	0.2658	0.035*	
C17	0.2245 (4)	0.6446 (2)	0.1325 (2)	0.0562 (6)	
H17A	0.3062	0.7380	0.1572	0.084*	
H17B	0.0697	0.6346	0.1452	0.084*	
H17C	0.2369	0.6346	0.0579	0.084*	
C18	0.5593 (3)	0.54184 (19)	0.17658 (15)	0.0365 (4)	
H18A	0.5814	0.5424	0.1024	0.055*	
H18B	0.6082	0.4608	0.2087	0.055*	
H18C	0.6443	0.6294	0.2099	0.055*	

### Atomic displacement parameters (Å^2^)

**Table:**

	*U*^11^	*U*^22^	*U*^33^	*U*^12^	*U*^13^	*U*^23^
S1	0.0300 (2)	0.0256 (2)	0.0255 (2)	0.00633 (14)	−0.00408 (14)	0.00327 (14)
F1	0.0410 (6)	0.0612 (7)	0.0347 (6)	0.0199 (5)	0.0117 (4)	−0.0051 (5)
O1	0.0268 (5)	0.0275 (5)	0.0268 (5)	0.0092 (4)	0.0047 (4)	0.0070 (4)
O2	0.0415 (7)	0.0373 (7)	0.0412 (7)	0.0215 (5)	0.0103 (5)	0.0049 (5)
O3	0.0502 (8)	0.0434 (7)	0.0398 (7)	0.0246 (6)	0.0185 (6)	0.0069 (5)
O4A	0.0480 (8)	0.0365 (7)	0.0234 (6)	0.0108 (6)	0.0003 (5)	0.0014 (5)
O4B	0.031 (3)	0.022 (9)	0.037 (10)	0.006 (7)	−0.004 (4)	0.018 (7)
C1	0.0246 (7)	0.0230 (7)	0.0245 (7)	0.0049 (5)	0.0009 (5)	0.0031 (5)
C2	0.0245 (7)	0.0216 (7)	0.0266 (7)	0.0035 (5)	0.0011 (6)	0.0024 (5)
C3	0.0311 (8)	0.0276 (7)	0.0270 (8)	0.0072 (6)	0.0051 (6)	0.0033 (6)
C4	0.0299 (8)	0.0261 (7)	0.0308 (8)	0.0049 (6)	0.0063 (6)	−0.0013 (6)
C5	0.0355 (9)	0.0305 (8)	0.0390 (9)	0.0108 (7)	0.0053 (7)	−0.0041 (7)
C6	0.0271 (7)	0.0216 (7)	0.0354 (8)	0.0064 (6)	0.0013 (6)	−0.0002 (6)
C7	0.0281 (8)	0.0250 (7)	0.0309 (8)	0.0073 (6)	0.0022 (6)	0.0053 (6)
C8	0.0234 (7)	0.0234 (7)	0.0272 (7)	0.0038 (5)	0.0033 (6)	0.0020 (6)
C9	0.0233 (7)	0.0227 (7)	0.0277 (7)	0.0050 (5)	−0.0003 (6)	0.0045 (6)
C10	0.0224 (7)	0.0245 (7)	0.0268 (7)	0.0028 (5)	0.0018 (5)	0.0016 (6)
C11	0.0322 (8)	0.0335 (8)	0.0285 (8)	0.0106 (6)	0.0027 (6)	0.0053 (6)
C12	0.0388 (9)	0.0406 (9)	0.0247 (8)	0.0093 (7)	0.0060 (7)	0.0051 (7)
C13	0.0273 (8)	0.0370 (8)	0.0285 (8)	0.0063 (6)	0.0045 (6)	−0.0073 (6)
C14	0.0284 (8)	0.0341 (8)	0.0325 (8)	0.0110 (6)	0.0005 (6)	−0.0001 (6)
C15	0.0284 (8)	0.0329 (8)	0.0284 (8)	0.0091 (6)	0.0037 (6)	0.0057 (6)
C16	0.0341 (8)	0.0233 (7)	0.0292 (8)	0.0061 (6)	0.0008 (6)	0.0002 (6)
C17	0.0508 (12)	0.0306 (9)	0.0863 (17)	0.0127 (8)	−0.0105 (11)	0.0137 (10)
C18	0.0325 (9)	0.0340 (9)	0.0409 (9)	0.0021 (7)	0.0025 (7)	0.0068 (7)

### Geometric parameters (Å, °)

**Table:**

S1—O4B	1.4716 (17)	C7—H7	0.9500
S1—O4A	1.4724 (13)	C9—C10	1.456 (2)
S1—C1	1.7783 (15)	C10—C15	1.398 (2)
S1—C16	1.8290 (16)	C10—C11	1.399 (2)
F1—C13	1.3623 (18)	C11—C12	1.382 (2)
O1—C8	1.3707 (18)	C11—H11	0.9500
O1—C9	1.3847 (17)	C12—C13	1.373 (2)
O2—C6	1.3712 (19)	C12—H12	0.9500
O2—C5	1.437 (2)	C13—C14	1.371 (2)
O3—C4	1.3740 (19)	C14—C15	1.384 (2)
O3—C5	1.428 (2)	C14—H14	0.9500
C1—C9	1.371 (2)	C15—H15	0.9500
C1—C2	1.443 (2)	C16—C18	1.510 (2)
C2—C8	1.393 (2)	C16—C17	1.521 (2)
C2—C3	1.412 (2)	C16—H16	1.0000
C3—C4	1.366 (2)	C17—H17A	0.9800
C3—H3	0.9500	C17—H17B	0.9800
C4—C6	1.398 (2)	C17—H17C	0.9800
C5—H5A	0.9900	C18—H18A	0.9800
C5—H5B	0.9900	C18—H18B	0.9800
C6—C7	1.371 (2)	C18—H18C	0.9800
C7—C8	1.395 (2)		
			
O4B—S1—O4A	104.0 (8)	O1—C9—C10	114.53 (13)
O4B—S1—C1	124.7 (6)	C15—C10—C11	118.20 (15)
O4A—S1—C1	107.07 (7)	C15—C10—C9	121.63 (14)
O4B—S1—C16	114.1 (7)	C11—C10—C9	120.13 (14)
O4A—S1—C16	107.44 (8)	C12—C11—C10	121.20 (15)
C1—S1—C16	98.56 (7)	C12—C11—H11	119.4
C8—O1—C9	107.04 (11)	C10—C11—H11	119.4
C6—O2—C5	105.72 (13)	C13—C12—C11	118.27 (15)
C4—O3—C5	106.07 (13)	C13—C12—H12	120.9
C9—C1—C2	107.40 (13)	C11—C12—H12	120.9
C9—C1—S1	127.89 (12)	F1—C13—C14	118.35 (15)
C2—C1—S1	124.56 (11)	F1—C13—C12	118.79 (15)
C8—C2—C3	120.03 (14)	C14—C13—C12	122.85 (15)
C8—C2—C1	105.09 (13)	C13—C14—C15	118.45 (15)
C3—C2—C1	134.88 (14)	C13—C14—H14	120.8
C4—C3—C2	114.84 (14)	C15—C14—H14	120.8
C4—C3—H3	122.6	C14—C15—C10	120.98 (15)
C2—C3—H3	122.6	C14—C15—H15	119.5
C3—C4—O3	126.86 (15)	C10—C15—H15	119.5
C3—C4—C6	123.73 (15)	C18—C16—C17	112.60 (15)
O3—C4—C6	109.37 (14)	C18—C16—S1	111.75 (12)
O3—C5—O2	107.97 (13)	C17—C16—S1	107.09 (12)
O3—C5—H5A	110.1	C18—C16—H16	108.4
O2—C5—H5A	110.1	C17—C16—H16	108.4
O3—C5—H5B	110.1	S1—C16—H16	108.4
O2—C5—H5B	110.1	C16—C17—H17A	109.5
H5A—C5—H5B	108.4	C16—C17—H17B	109.5
O2—C6—C7	127.12 (15)	H17A—C17—H17B	109.5
O2—C6—C4	109.74 (14)	C16—C17—H17C	109.5
C7—C6—C4	123.13 (14)	H17A—C17—H17C	109.5
C6—C7—C8	113.00 (14)	H17B—C17—H17C	109.5
C6—C7—H7	123.5	C16—C18—H18A	109.5
C8—C7—H7	123.5	C16—C18—H18B	109.5
O1—C8—C2	110.64 (13)	H18A—C18—H18B	109.5
O1—C8—C7	124.08 (13)	C16—C18—H18C	109.5
C2—C8—C7	125.27 (14)	H18A—C18—H18C	109.5
C1—C9—O1	109.82 (13)	H18B—C18—H18C	109.5
C1—C9—C10	135.63 (14)		
			
O4B—S1—C1—C9	−43.3 (10)	C3—C2—C8—C7	0.3 (2)
O4A—S1—C1—C9	−164.61 (14)	C1—C2—C8—C7	−179.36 (14)
C16—S1—C1—C9	84.09 (15)	C6—C7—C8—O1	−178.87 (13)
O4B—S1—C1—C2	131.6 (10)	C6—C7—C8—C2	0.3 (2)
O4A—S1—C1—C2	10.31 (15)	C2—C1—C9—O1	0.33 (16)
C16—S1—C1—C2	−100.99 (13)	S1—C1—C9—O1	175.95 (10)
C9—C1—C2—C8	−0.13 (16)	C2—C1—C9—C10	−178.34 (16)
S1—C1—C2—C8	−175.93 (11)	S1—C1—C9—C10	−2.7 (3)
C9—C1—C2—C3	−179.69 (16)	C8—O1—C9—C1	−0.41 (16)
S1—C1—C2—C3	4.5 (3)	C8—O1—C9—C10	178.57 (12)
C8—C2—C3—C4	−0.7 (2)	C1—C9—C10—C15	0.4 (3)
C1—C2—C3—C4	178.84 (16)	O1—C9—C10—C15	−178.21 (13)
C2—C3—C4—O3	−177.24 (15)	C1—C9—C10—C11	178.06 (17)
C2—C3—C4—C6	0.6 (2)	O1—C9—C10—C11	−0.6 (2)
C5—O3—C4—C3	−175.24 (16)	C15—C10—C11—C12	1.7 (2)
C5—O3—C4—C6	6.70 (18)	C9—C10—C11—C12	−176.06 (15)
C4—O3—C5—O2	−10.48 (18)	C10—C11—C12—C13	0.1 (3)
C6—O2—C5—O3	10.29 (17)	C11—C12—C13—F1	177.04 (15)
C5—O2—C6—C7	175.46 (16)	C11—C12—C13—C14	−1.9 (3)
C5—O2—C6—C4	−6.21 (17)	F1—C13—C14—C15	−177.15 (14)
C3—C4—C6—O2	−178.41 (15)	C12—C13—C14—C15	1.8 (3)
O3—C4—C6—O2	−0.28 (18)	C13—C14—C15—C10	0.1 (2)
C3—C4—C6—C7	0.0 (3)	C11—C10—C15—C14	−1.8 (2)
O3—C4—C6—C7	178.12 (14)	C9—C10—C15—C14	175.91 (14)
O2—C6—C7—C8	177.70 (14)	O4B—S1—C16—C18	−159.6 (8)
C4—C6—C7—C8	−0.4 (2)	O4A—S1—C16—C18	−44.95 (14)
C9—O1—C8—C2	0.32 (16)	C1—S1—C16—C18	66.05 (13)
C9—O1—C8—C7	179.57 (14)	O4B—S1—C16—C17	−35.9 (8)
C3—C2—C8—O1	179.52 (13)	O4A—S1—C16—C17	78.81 (15)
C1—C2—C8—O1	−0.12 (16)	C1—S1—C16—C17	−170.20 (14)

### Hydrogen-bond geometry (Å, °)

**Table:**

*D*—H···*A*	*D*—H	H···*A*	*D*···*A*	*D*—H···*A*
C5—H5B···O4A^i^	0.99	2.27	3.231 (2)	163
C18—H18A···O4A^ii^	0.98	2.49	3.354 (2)	147

Symmetry codes: (i) −*x*+1, −*y*, −*z*; (ii) −*x*+1, −*y*+1, −*z*.
